# Nebula

**DOI:** 10.1007/s10912-025-09946-5

**Published:** 2025-04-15

**Authors:** Natalie Mera Ford

**Affiliations:** Swarthmore College, Swarthmore, USA

If the moon can wane

as well as wax, if the iris

in bloom can contract

after extravagant dilation,

if a cloud of astral dust

can some century suck in

the atoms that once hovered

and slid, ghostly, over our slip

of the galaxy,

then this blur of pigment,

this melanin stretching a train

of dark ivy across my skin,

can also reverse its growth—

can draw back in an unleafing,

in a lunar non-eclipse,

in a micro big crunch

with all the outbreaking, heart-

breaking particles

willed to implode.

## Data Availability

No datasets were generated or analysed during the current study.

